# Expression of *uPAR* mRNA in peripheral blood is a favourite marker for metastasis in gastric cancer cases

**DOI:** 10.1038/sj.bjc.6604806

**Published:** 2008-12-02

**Authors:** Y Kita, T Fukagawa, K Mimori, Y Kosaka, K Ishikawa, T Aikou, S Natsugoe, M Sasako, M Mori

**Affiliations:** 1 Department of Surgery, Medical Institute of Bioregulation, Kyushu University, 4546, Tsurumihara, Beppu 874-0838, Japan; 2 Department of Surgical Oncology and Digestive Surgery, Field of Oncology, Course of Advanced Therapeutics, Kagoshima University, Graduate School of Medical and Dental Science, Kagoshima University, 8-35-1, Sakuragaoka, Kagoshima 890-8520, Japan; 3 Gastric Surgery Division, National Cancer Center Hospital, 5-1-1 Tsukiji, Chuo-ku 104-0045, Japan

**Keywords:** gastric cancer, circulating tumour cells, peripheral blood, bone marrow, urokinase-type plasminogen activator receptor (*uPAR*), metastasis

## Abstract

Urokinase-type plasminogen activator receptor (*uPAR*) plays a central role in the plasminogen activation cascade and participates in extracellular matrix degradation, cell migration and invasion. We evaluated the expression level of *uPAR* mRNA and the presence of isolated tumour cells (ITCs) in bone marrow (BM) and peripheral blood (PB) in gastric cancer patients and clarified its clinical significance. We assessed specific *uPAR* mRNA expression by quantitative real-time reverse transcriptase- polymerase chain reaction (RT–PCR) in BM and PB in 846 gastric cancer patients as well as three epithelial cell markers, carcinoembryonic antigen (*CEA*), cytokeratin *(CK)-19* and *CK-7*. The *uPAR* mRNA expression in bone marrow and peripheral blood expressed significantly higher than normal controls (*P*<0.0001). The *uPAR* mRNA in BM showed concordant expression with the depth of tumour invasion, distant metastasis, and the postoperative recurrence (*P*=0.015, 0.044 and 0.010, respectively); whereas in PB, we observed more intimate significant association between *uPAR* expression and clinicopathologic variables, such as depth of tumour invasion, the distant metastasis, the venous invasion and the clinical stage (*P*=0.009, 0.002, 0.039 and 0.008, respectively). In addition, the *uPAR* mRNA expression in PB was an independent prognostic factor for distant metastasis by multivariate analysis. We disclosed that it was possible to identify high-risk patients for distant metastasis by measuring *uPAR* mRNA especially in peripheral blood at the timing of operation in gastric cancer patients.

The presence of isolated tumour cells (ITCs) in bone marrow (BM) and peripheral blood (PB) is missed by conventional imaging system, and ITCs expected to be a determinant factor of subsequent formation of micrometastasis. The search for ITCs in patients with curatively resected tumours is of considerable importance, because early dissemination of tumour cells is one of the leading causes of relapse at the distant site and of death from cancer (Hellman, 1997). In spite of large number of studies to determine the clinicopathologic significance of ITCs in human solid carcinomas, much efforts have been made and found no definitive evidence to conclude these controversial issues, such as the way to identify ITCs and the appropriate biologic marker to predict the metastatic ability of gastric cancer cells (Heiss *et al*, 1995, 1997, 2002; Jauch *et al*, 1996; Hardingham *et al*, 2000; Lecomte *et al*, 2002; Natsugoe *et al*, 2003; Ikeguchi and Kaibara, 2005).

To identify ITCs in BM or PB, a bunch of molecular targets, such as CEA, CK7, CK18, CK18, CK19 and MAGE families have been applied and examined whether those genes were applicable to detect ITCs. Above all candidates, *CEA* and cytokeratin *(CK),* epithelial cell surface markers were frequently used to be applied to detect ITCs instead of the direct detection of cancer cells in those ITC studies. However, our recent study disclosed that ITCs in BM and PB from gastric cancer could not be specified by the presence of CEA and/or CKs by RT–PCR, because those genes were detected ubiquitously among stages of 810 patients of gastric cancer. Therefore, we must identify a favourite marker to detect ITCs specifically as well as to predict metastasis precisely. In the current study, we focused on urokinase-type plasminogen activator receptor (*uPAR*) gene as a target molecule to detect isolated tumour cells in blood and bone marrow. This is because that in gastric cancer, several reports showed clearly that uPAR expression in bone marrow (BM) is one of the useful prognostic marker by immunohistochemistry (Heiss *et al*, 1995, 2002; Allgayer *et al*, 1997). However, there are no earlier reports of the clinical significance of the gene expression level of *uPAR* with quantitative RT–PCR assay that enabled us to examine objectively and repeatedly. Therefore, the aim of the current study was to evaluate the expressions of *uPAR* mRNA in bone marrow and peripheral blood in more than 800 cases of gastric cancer and to define its clinicopathologic and prognostic significance in gastric cancer patients.

## Materials and methods

### Gastric cancer cases

A total of 846 gastric cancer patients who underwent surgical treatment in the National Cancer Center Hospital, Japan from 2001 to 2004 were studied. Clinical stages and pathological features of primary tumours were defined according to the classification of the International Union Against Cancer (Sobin and Fleming, 1997). There were 567 male and 279 female patients with average age 61.5, and a range of 27–87 years. None of these patients underwent endoscopic mucosal resection or palliative resection. Written informed consent had been obtained from all patients.

### Normal controls

For normal negative controls, both peripheral blood and bone marrow were collected from 25 patients having no malignancy from April 2000 to March 2003. This group included 16 cases of gallstone, three cases of common bile duct stone, and six cases of incisional hernia. We extracted BM from the sternum of patients without malignancies but had operations under general anaesthesia.

### Bone marrow and blood samples

Aspiration of both BM and PB was conducted immediately prior to operation under general anaesthesia. The aspirated BM was obtained from the sternum using a bone marrow aspiration needle (MDTECH, Gainesville, FL, USA). Peripheral blood was obtained through a venous catheter. The 1 ml of both BM and PB samples was discarded to avoid the contamination of the epidermal cells (Iinuma *et al*, 2006). Each 1 ml sample of BM and PB was immediately mixed with 4 ml of ISOGEN-LS (Nippon Gene, Toyama, Japan) and stored at −80°C until RNA extraction.

### Total RNA extraction and first strand cDNA synthesis

Total RNA was according to the ISOGEN-LS manufacturer's protocols. All the clinical samples obtained in National Cancer Center Hospital were sent to our institute. The reverse transcriptase reaction (RT) was performed as described earlier (Mori *et al*, 1995; Masuda *et al*, 2002). The first strand cDNA was synthesized from 2.7 *μ*g of total RNA in a 30 *μ*l reaction mixture containing 5 *μ*l 5 × RT reaction buffer (BRL, Gaithersburg, MD, USA), 200 *μ*M dNTP, 100 *μ*M solution of random hexadeoxynucleotide mixture, 50 units of Rnasin (Promega, Madison, WI, USA), 2 *μ*l of 0.1 M dithiothreitol, and 100 U of Molony leukaemia virus RT (BRL). The mixture was incubated at 37°C for 60 min, heated to 95°C for 10 min, and then chilled on ice.

### Primers and probes for detecting ITCs and *uPAR* expression

Quantitative RT–PCR methodology was designed to optimise the specificity and fidelity of the assay. The Kyushu University group had previously investigated the expression of seven representative molecular markers (carcinoembryonic antigen (*CEA*), *CK-7*, *CK-18*, *CK-19*, *CK-20*, mammaglobin and mucin (*MUC*)-1) in 27 cancers and eight non-epithelial cell lines using quantitative RT–PCR. The expression levels of *CK-7* and *CK-19* showed high sensitivity and specificity for the identification of gastric cancer cells in comparison with the other markers (Masuda *et al*, 2005). Those epithelial cell markers have been widely used as target genes for the detection of ITCs (Mori *et al*, 1996, 1998; Yamaguchi *et al*, 2000; Bessa *et al*, 2003; Sadahiro *et al*, 2005). Thus, *CEA*, *CK-7*, *CK-19* and *GAPDH* were studied by quantitative RT–PCR in all 846 patients (Mimori *et al*, 2008). Isolated tumour cells were considered to be ‘present’ when any single marker was positive. The reverse transcriptase reaction was performed as described elsewhere (Mimori *et al*, 2008). We performed real-time quantitative RT–PCR using a LightCycler instrument (Roche Diagnostics, Manheim, Germany) to detect ITCs in peripheral blood and/or bone marrow as the previous study.

Moreover, a *uPAR*-specific oligonucleotide primer was designed as follows: sense, 5′-TGAATCAATGTCTGGTAGC-3′ and antisense, 5′-TGGTTACAGCCACTTTTAGT-3′. The donor and acceptor probe sequences for *uPAR* identification were 5′-GCTATATGGTAAGAGGCTGTGCAACCGCCT-fluorescein and 5′-LC-Red640-AATGTGCCAACATGCCCACCTGGG T -phosphorylation. Besides, *uPA* primers were as follows: forward primer; CTGTGACTGTCTAAATGGAGG; and the reverse primer; GACGATGTAGTCCTCCTTCTT (Nielsen *et al*, 2004).

Preliminary trials were undertaken to assure that results were accurate and reliable. We utilised highly specific hybridisation probes and primers to maintain high specificity for target genes and thereby reduce false positive outcomes.

### RT–PCR conditions

PCR amplification was performed using a quantitative fluorescence LightCycler™ (Roche Diagnostics, Mannheim, Germany) in a 20 *μ*l reaction mixture containing 2 *μ*l of LightCyclert FastStart DNA Master Hybridisation Probes (Roche, Diagnostics, Tokyo, Japan), 4.0 *μ*l MgCl_2_, 0.3 *μ*M sense and antisense primers, 0.2 *μ*M fluorescent probe, 0.2 *μ*M LC-Red probe and 5 *μ*l of undiluted template cDNA in LightCyclert capillaries (Roche Diagnostics, Tokyo, Japan). The amplification of *CEA* profile consisted of one cycle at 95°C for 10 min (denaturation) followed by 45 cycles of 95°C for 15 s, 56°C for 15 s and 72°C for 13 s. The amplification of *CK-7* profile consisted of one cycle at 95°C for 10 min (denaturation) followed by 50 cycles of 95°C for 10 s, 60°C for 12 s and 72°C for 10 s. The amplification of *CK-19* profile consisted of one cycle at 95°C for 10 min (denaturation) followed by 45 cycles of 95°C for 10 s, 60°C for 15 s and 72°C for 16 s. The amplification of *uPAR* profile consisted of one cycle at 95°C for 10 min (denaturation) followed by 40 cycles of 95°C for 10 s, 62°C for 15 s and 72°C for 8 s. The amplification of *GAPDH* profile consisted of one cycle at 95°C for 10 min (denaturation) followed by 40 cycles of 95°C for 15 s, 60°C for 15 s and 72°C for 13 s. The amplification of uPA was as follows: 5 min at 94°C, 27 cycles of 1 min at 94°C, 1 min at 56°C, 1 min at 72°C, then 10 min at 72°C (Nielsen *et al*, 2004). Real-time PCR was monitored by measuring fluorescent signals at the end of the annealing phase for each cycle. All primers and probes were synthesized and purified by reverse phase high-performance liquid chromatography and the optimal reagent concentrations and PCR cycling conditions were established and each run of RT–PCR included positive controls synthesized from plasmids by the Nippon Gene Research Laboratories (Sendai, Japan). Real-time RT–PCR assays were repeated in triplicate and adapted the mean value. Quantification data were analysed using the Light-Cycler™ software (Roche Diagnostics, Tokyo, Japan).

### Data analysis

A standard curve was prepared with 200–20 000 copies of purified plasmids containing *CEA*, *CK-7*, *CK-19*, *uPAR* and *GAPDH*. After proportional baseline adjustment, the fit point method was employed to determine the cycle in which the log-linear signal was first distinguishable from the baseline, and then that cycle number was used as a crossing-point value (Marutsuka *et al*, 2003). The standard curve was produced by measuring the crossing point of each standard value and plotting them against the logarithmic value of concentrations. Those concentrations were calculated by plotting their crossing points against the standard curve.

Expression of CEA, CK-7, CK-19 and *uPAR* was adjusted in each case for GAPDH expression. We set cutoff values for those expression ratios (*CEA/GAPDH*, *CK-7/GAPDH*, *CK-19/GAPDH*, *uPAR/GAPDH*) as the highest value for each marker in 25 normal controls. We distributed high level as ‘positive’ and low level as ‘negative’ than the cutoff value. For continuous variables, the data were expressed as the mean±s.d. Statistical analysis of group differences was performed using the *χ*^2^ test and the Student's *t*-test. Logistic regression model was used to identify the independent predictors of distant metastasis. All tests were analysed using JMP software (SAS Institute Inc., Cary, NC, USA). Statistical significance was determined as *P*-value from two-sided tests of less than 0.05.

## Results

### Expression of *uPAR* mRNA in BM and PB of gastric cancer patients

**Figure 1:**
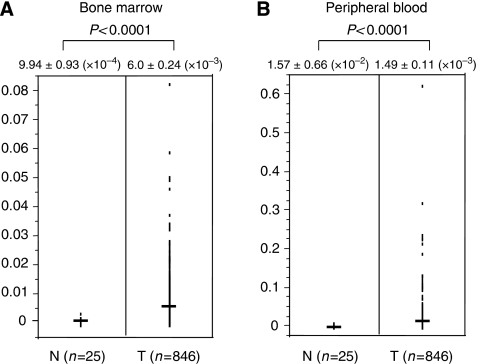
Comparison of *uPAR* mRNA expression between control gastric cancer patients in bone marrow (**A**) and peripheral blood (**B**). Horizontal lines indicate the mean expression levels in control and gastric cancer patients. In bone marrow and peripheral blood, the value of the *uPAR* expression of gastric cancer patients was significantly higher (*P*<0.0001) than those of control cases. The *P*-value was calculated by a student's *t*-test.

In BM, the value of the *uPAR* expression of gastric cancer patients (6.0±0.24 ( × 10^−3^)) was significantly higher (*P*<0.0001) than those of control cases (9.94±0.93 ( × 10^−4^)), as shown in Figure 1. In PB, the value of the *uPAR* expression of gastric cancer patients (1.49±0.11 ( × 10^−2^)) was also significantly higher (*P*<0.0001) than those of control cases (1.57±0.66 ( × 10^−3^)).

### Clinical magnitude of *uPAR* expression in gastric cancer patients

The correlations between the results for the *uPAR* mRNA level and clinicopathologic factors are summarised in Table 1. Using each cutoff value, 431 (50.9%) and 404 (47.8%) of 846 patients were estimated to be positive for *uPAR* mRNA in BM and PB, respectively.

In BM, the significantly higher population of the *uPAR* mRNA-positive cases belongs to the following clinical subgroups: invasion deeper than the muscularis propria (*P*=0.015), perioperative overt metastases (including liver and/or lung and/or distant lymph node metastasis, *P*=0.044) and postoperative recurrence (*P*=0.010).

In PB, the higher expression was observed significantly in subgroup invasion deeper than the muscularis propria (*P*=0.009), perioperative overt metastases (*P*=0.002), venous invasion (*P*=0.039), and the clinical stage (*P*=0.008).

### Multivariate analysis for distant metastasis

Univariate and multivariate logistic regression analyses were performed on cases with distant metastasis (Table 2). Univariate regression analysis showed that the following factors were significantly associated with distant metastasis: histological grade, tumour size, lymph node metastasis, lymphatic invasion, venous invasion and *uPAR* mRNA expressions in BM and PB (*P*<0.05), respectively. Multivariate regression analysis indicated that *uPAR* mRNA high expression group in PB (relative risk (RR); 1.85, 95 % confidence interval (CI); 1.08–3.23, *P*=0.03) was an independent predictor for distant metastasis next to the incidence lymph node metastasis (RR; 6.50, 95 % CI; 2.99–15.77, *P*<0.0001) and depth of tumour invasion (RR; 28.2, 95% CI; 14.3–70.0, *P*<0.0001).

### The comparison of *uPA* and *uPAR* expression in representative gastric cancer cases

The potential importance of uPAR for the development of minimal residual disease in solid cancer has been focused on for recent two decades; however, several studies revealed that the relevance of a ligand for *uPAR*, *uPA* in mediating tumour-associated proteolysis, invasion and metastasis together with *uPAR* expression (Andreasen *et al*, 2000). We examined *uPA* expression in bone marrow and peripheral blood in representative 83 cases of gastric cancer, including 18 cases of liver and/or lung metastasis, and 12 cases of recurrence of disease among 846 cases of gastric cancer. As we showed in Table 3, we found that *uPA* expression in bone marrow from gastric cancer is correlated with the incidence of lymph node metastasis and recurrence of gastric cancer cases as *uPAR* expression.

### The clinical significance of both positive ITCs and positive *uPAR* expression

We identified 66 cases out of 846 (7.8%) that were positive for expression of CEA in bone marrow, whereas 108 (12.7%) were positive in peripheral blood. As for CK-7, 71 patients (8.4%) and 147 cases (20.6%) were positive in bone marrow and in peripheral blood, respectively. Cytokeratin-19 expression was detected in bone marrow in 153 cases (18.1%) and in 251 cases (29.7%) in peripheral blood. Gastric cancer cases with positive expression of any one of the three markers were defined as ITC-positive in bone marrow or peripheral blood. As outlined above, ITCs were detected in 260 (30.7%) cases in bone marrow and 417 (49.3%) cases in peripheral blood.

Moreover, we extracted 126 cases (14.9%) in BM and 200 cases (23.6%) in PB of positive ITCs and positive *uPAR* expression cases. Table 4 shows the clinicopathologic data and positive ITCs and positive uPAR cases from the 846 gastric cancer patients. In BM, the incidence of lymph node metastasis was significantly higher (*P*=0.012) in the both positive group (65 of 126, 51.6%) than in the other group (285 of 720, 39.6%), and the incidence of lymphatic invasion was significantly higher (*P*=0.009) in the both positive group (67 of 126, 53.2%) than in the other group (293 of 720, 40.7%). Moreover, the clinical stage was significantly higher (*P*=0.024) in the both positive group (42 of 126, 33.3%) than in the other group (170 of 720, 23.4%). In PB, there was no significance between the both positive group and the other group.

## Discussion

In this study, we examined the clinicopathologic significance of *uPAR* expression in BM and PB in 846 cases of gastric cancer, and found that the depth of tumour invasiveness in the primary tumour was significantly higher in cases of *uPAR* (+) in BM and/or PB than *uPAR*(−) cases. In addition, we disclosed an evidence of the concordant relationship between the venous permeation in primary cancer and the incidence of *uPAR* expression. In spite of the strong association between tumour invasiveness, the presence of *uPAR* in PB was uncovered to be much more intimate to the incidence of metastasis and could be an independent prognostic indicator for hematogenous metastasis. Therefore, we consider that *uPAR* might play a consecutive role in cancer cells to invade into vessels and/or invading into the metastatic site. Furthermore, the clinical relevance of *uPAR* in bone marrow was observed with the incidence of recurrence, not with the synchronous distant metastasis. There was a possible explanation which is as follows, Kook *et al* (1994) reported that *uPAR* can play a role in tumour cell dormancy. They reported that a *uPAR*-antisense strategy in a human squamous carcinoma cell line resulting in a significant reduction of *uPAR* gene expression, induced tumour cell dormancy in their study. Besides, Laufs *et al* (2006) described this point in the review concerning *uPAR*. Therefore, the abundant expression of *uPAR* in bone marrow might indicate the presence of many dormant cells giving rise to recurrence in the future.

In an earlier study by Jauch *et al* (1996) uPAR is a glycosyl-phosphatidylinositol-anchored glycoprotein localised on the outer layer of the plasma membrane of cells, and it binds to its specific ligands such as urokinase-type plasminogen activator (uPA). In this study, we confirmed the concordant relationship between *uPAR* and *uPA* in bone marrow and peripheral blood indicating that both proteins have a synergistic role with each other in lymph node metastasis and recurrence of gastric cancer. However, uPAR activation ultimately leads to degradation of the extracellular matrix and fascinates cellular movement for tumour cells, which appears to be necessary for diverse functions including local invasion and metastasis of tumour cells (Heiss *et al*, 1997).

Then, the second point is what is the origin of cells expressing *uPAR* gene in BM and especially in PB. As a matter of fact, Heiss *et al* (1995, 1997, 2002) reported that the gastric cancer patients with cells with uPAR protein expression by immunocytology showed significantly poorer prognosis than cases without uPAR expression by Kaplan–Meier analysis in the previous study (Jauch *et al*, 1996; Hardingham *et al*, 2000). They confirmed that uPAR protein expressing cells on the surface cells was a cancer cell by immunocytological study. Their study strongly supported the current study by quantitative RT–PCR assay that the detected *uPAR* expression by RT–PCR should be originally from gastric cancer cells, and gastric cancer patients with cancer cells with the invasive ability especially in PB. Moreover, we additionally disclosed that cancer patients with simultaneous expression uPAR and epithelial cell markers, *CEA*, *CK19* and *CK7* showed a relatively poorer prognosis than ITCs alone. Gastric cancer cell isolated from primary cancer is circulating in the peripheral blood and bone marrow ubiquitously among whole stages (Mimori *et al*, 2008); however, isolated cancer cells with several potential abilities must be required to form metastasis. According to this study, we concluded that *uPAR*-expressing isolated tumour cells are important in the determination of recurrence through lymph node metastasis.

On the contrary to the hypothesis of the origin of uPAR expression in cancer cells, several studies have uncovered findings of the uPAR from host side cells in BM or PB from cancer patients. Pyke *et al* (1993) reported the abundant expression of uPAR in macrophage (Dubuisson *et al*, 2000), and Sugai *et al* (2004) disclosed that advances in gastric cancer cases indicated the activation of inflammatory cytokines, such as IL-10 and IL-12 from macrophages. Furthermore, Hildenbrand *et al* (1998) mentioned that the abundant expression of uPAR was observed in endothelial cells, which has been recently really focused on as the key player for the initial development of metastasis. Mancuso *et al* (2001) reported that the number of circulating endothelial cells (CECs) in PB from cancer patients are more than that in healthy volunteers (Beerepoot *et al*, 2004). Asahara *et al* (1997) reported that bone marrow-derived endothelial cell progenitor cells were disseminated to the neovascularisation of the cellular surface of malignant cells (Peters *et al*, 2005). In addition, EPC-specific gene, Id-1, was reported to be identified and its consecutive role for metastasis has been reported in the recent study. Therefore, we considered that the presence of CEC or EPC in PB should be important to form metastasis, and our current study elucidated the role of uPAR especially in PB as the independent marker for metastasis.

In this study, we concluded that the RT–PCR assay for *uPAR* expression in PB can be one of the favourite tumour markers to predict DFS in gastric cancer outpatients. Then, we disclosed the abundant expression of uPAR in gastric cancer cases with invasion and with venous invasion abilities. Earlier Heiss *et al* distinctively disclosed that uPAR expression in BM and PB in gastric cancer is originally from cancer cells themselves (Heiss *et al*, 1995). However, as the clinicopathologic significance and the predictive role for metastasis is much more consecutive in uPAR in PB than in BM, uPAR might be originally expressed in endothelial (progenitor) cells as the host side reaction in gastric cancer patients. Further study will be required to address this controversial issue.

## Figures and Tables

**Table 1:** Relationship between uPAR expression in bone marrow and peripheral blood and clinicopathologic findings

		**uPAR expression in bone marrow**			**uPAR expression in peripheral blood**		
		**Positive**	**Negative**		**Positive**	**Negative**	
**Total**	**846**	***n*=431 (50.9%)**	***n*=415 (49.1%)**	***P*-value**	***n*=404 (47.8%)**	***n*=442 (52.2%)**	***P*-value**
Age (mean±s.d.^a^)		60.4±0.55	62.7±0.56	NS	61.5±0.57	61.6±0.55	NS
*Gender*				0.233			0.688
Male	567	297 (69.1)	270 (65.2)		268 (66.5)	299 (67.8)	
Female	279	134 (30.9)	145 (34.8)		136 (33.5)	143 (32.2)	
Histology				0.127			0.174
Differentiated	188	105 (24.4)	83 (20.0)		98 (24.3)	90 (20.4)	
Undifferentiated	658	326 (75.6)	332 (80.0)		306 (75.7)	352 (79.6)	
pT				0.015			0.009
pT1/T2	657	320 (74.3)	337 (81.2)		298 (73.8)	359 (81.2)	
pT3/T4	189	111 (25.7)	78 (18.8)		106 (26.2)	83 (18.8)	
pN				0.707			0.072
pN0	496	250 (58.0)	246 (59.3)		224 (55.5)	272 (61.5)	
pN1	350	181 (42.0)	169 (40.7)		180 (44.5)	170 (38.5)	
pM (Distant metastasis^a^)				0.044			0.002
pM0	743	369 (85.6)	374 (90.1)		340 (84.2)	403 (91.2)	
pM1	103	62 (14.4)	41 (9.9)		64 (15.8)	39 (8.8)	
Postoperative recurrence				0.01			0.115
Non recurrence	833	420 (97.5)	413 (99.5)		395 (97.8)	438 (99.1)	
Recurrence	13	11 (2.5)	2 (0.5)		9 (2.2)	4 (0.9)	
Lymphatic invasion				0.617			0.16
Negative	486	244 (58.3)	242 (58.3)		222 (55.0)	264 (59.7)	
Positive	360	187 (56.6)	173 (41.7)		182 (45.0)	178 (40.3)	
Venous invasion				0.425			0.039
Negative	702	362 (83.9)	340 (81.9)		324 (80.2)	378 (85.5)	
Positive	144	69 (16.1)	75 (18.1)		80 (19.8)	64 (14.5)	
Stage				0.081			0.008
I, II	634	312 (72.4)	322 (77.6)		286 (70.8)	348 (78.7)	
III, IV	212	119 (27.6)	93 (22.4)		118 (29.2)	94 (21.3)	

a Gastric cancer case with liver (H1) and/or lung metastasis (M1), cytology positive of peritoneal washes (CY1), peritoneal dissemination (P), and lymph node metastasis in the distant region (N3).

**Table 2:** Univariate and multivariate analysis for distant metastasis (logistic regression model)

	**Univariate analysis**			**Multivariate analysis**		
	**RR**	**95% CI**	***P*-value**	**RR**	**96% CI**	***P*-value**
Histological grade (Differentiated/undifferentiated)	2.35	1.31–2.35	<0.0001	0.58	0.24–1.45	0.239
pT (Depth of tumour invasion)	49.91	27.36–99.07	<0.0001	28.17	14.30–60.95	<0.0001
pN (Lymph node metastasis)	22.78	11.56–51.49	<0.0001	6.5	2.99–15.77	<0.0001
Lymphatic invasion	12.12	6.89–23.12	<0.0001	—	—	—
Venous invasion	4.55	2.91–7.10	<0.0001	1.61	0.90–2.88	0.106
*uPAR* mRNA expression in bone marrow	1.53	1.01–2.35	0.046	—	—	—
*uPAR* mRNA expression in peripheral blood	1.95	1.28–2.99	0.002	1.85	1.08–3.23	0.0207

**Table 3:** Clinicopathologic significance of uPAR and uPA expressions in bone marrow (BM) and peripheral blood (PB) from gastric cancer cases

		**uPAR-BM**			**uPAR-PB**			**uPA-BM**			**uPA-PB**		
	***n***	**Mean**	**s.d.**	***P*-value**	**Mean**	**s.d.**	***P*-value**	**Mean**	**s.d.**	***P*-value**	**Mean**	**s.d.**	***P*-value**
*ly*				NS			NS			NS			NS
−	58	8.9E-03	6.2E-03		1.4E-02	8.1E-03		2.1E-02	1.3E-02		2.4E-03	2.2E-03	
+	25	1.1E-02	1.1E-02		1.3E-02	9.5E-03		2.2E-02	1.8E-02		3.1E-03	7.3E-03	
													
*v*				NS			NS			NS			NS
−	65	9.7E-03	7.8E-03		1.3E-02	8.3E-03		2.1E-02	1.2E-02		2.2E-03	2.1E-03	
+	18	9.3E-03	9.0E-03		1.5E-02	9.2E-03		2.4E-02	2.0E-02		4.0E-03	8.6E-03	
													
*n*				**0.0006**			NS			**0.0002**			NS
−	62	7.9E-03	5.4E-03		1.3E-02	8.5E-03		1.8E-02	1.2E-02		2.8E-03	5.1E-03	
+	21	1.5E-02	1.2E-02		1.3E-02	8.7E-03		3.1E-02	1.7E-02		2.1E-03	1.3E-03	
													
*cy*				NS			NS			NS			NS
−	71	9.2E-03	7.9E-03		1.3E-02	8.4E-03		2.1E-02	1.5E-02		2.5E-03	4.6E-03	
+	16	1.3E-02	8.5E-03		1.5E-02	8.1E-03		2.6E-02	1.5E-02		3.2E-03	2.6E-03	
													
*P*				NS			NS			NS			NS
−	80	9.5E-03	7.9E-03		1.3E-02	8.4E-03		2.1E-02	1.5E-02		2.6E-03	4.5E-03	
+	8	1.3E-02	9.4E-03		1.4E-02	7.6E-03		2.6E-02	1.6E-02		2.7E-03	1.9E-03	
													
*met*				NS			NS			NS			**0.0265**
−	70	9.2E-03	7.9E-03		1.3E-02	8.2E-03		2.0E-02	1.3E-02		2.1E-03	1.9E-03	
+	18	1.2E-02	8.7E-03		1.6E-02	8.4E-03		2.8E-02	1.9E-02		4.6E-03	8.7E-03	
													
*rec*				**0.0072**			NS			**0.0409**			NS
−	76	8.9E-03	6.6E-03		1.4E-02	8.6E-03		2.0E-02	1.5E-02		2.6E-03	4.6E-03	
+	12	1.6E-02	1.3E-02		1.1E-02	5.9E-03		3.0E-02	1.3E-02		2.2E-03	1.5E-03	

cy=cytology of peritoneal washes; ly=lymphatic permiation; met=liver and/or lung metastasis; n=lymph node metastasis; p=peritoneal dissemination; rec=recurrence; s.d.=standard deviation; v=vascular permiation. Significant differences (*P*<0.05) were described in bold letters.

**Table 4:** Relationship between epithelial marker and uPAR expression in bone marrow and peripheral blood and clinicopathologic findings

		**Epithelial marker and uPAR expression in BM**			**Epithelial marker and uPAR expression in PB**		
		**Both positive**	**Others**		**Both positive**	**Others**	
	**Total (n=846)**	***n*=126**	***n*=720**	***P*-value**	***n*=200**	***n*=646**	***P*-value**
Age (mean±s.d.^*^)		60.9±0.97	61.6±0.43	NS	60.8±0.81	61.7±0.45	NS
*Gender*				0.403			
Male	567	88 (69.8)	479 (66.5)		130 (65.0)	437 (67.6)	0.526
Female	279	38 (30.2)	241 (33.5)		70 (35.0)	209 (32.4)	
							
*Histology*				0.645			
Differentiated	188	30 (23.8)	158 (21.9)		45 (22.5)	143 (22.1)	0.914
Undifferentiated	658	96 (76.2)	562 (78.1)		155 (77.5)	503 (77.9)	
							
*pT*				0.183			
pT1/T2	657	92 (73.0)	565 (78.5)		147 (73.5)	510 (79.0)	0.11
pT3/T4	189	34 (27.0)	155 (21.5)		53 (26.5)	136 (21.0)	
							
*pN*					0.012		
pN0	496	61 (48.4)	435 (60.4)		112 (56.0)	384 (59.4)	0.389
pN1	350	65 (51.6)	285 (39.6)		88 (44.0)	262 (40.6)	
							
*pM (Distant metastasis)*				0.108			
pM0	743	105 (83.3)	638 (88.6)		170 (85.0)	573 (88.7)	0.171
pM1	103	21 (16.7)	82 (11.4)		30 (15.0)	73 (11.3)	
							
*Postoperative recurrence*				0.145			
Non recurrence	833	122 (96.8)	711 (98.8)		197 (98.5)	636 (98.5)	0.961
Recurrence	13	4 (3.2)	9 (1.2)		3 (1.5)	10 (1.5)	
							
*Lymphatic invasion*				0.009			
Negative	486	59 (46.8)	427 (59.3)		117 (58.5)	369 (57.1)	0.73
Positive	360	67 (53.2)	293 (40.7)		83 (41.5)	277 (42.9)	
							
*Venous invasion*				0.102			
Negative	702	98 (77.8)	604 (83.9)		162 (81.0)	540 (83.6)	0.399
Positive	144	28 (22.2)	116 (16.1)		38 (19.0)	106 (16.4)	
							
*Stage*				0.024			
I, II	634	84 (66.7)	550 (76.4)		141 (70.5)	493 (76.3)	0.101
III, IV	212	42 (33.3)	170 (23.4)		59 (29.5)	153 (23.7)	

^*^s.d.=standard deviation.
